# Establishment of a Single-Oocyte Culture System for Pigs and Its Validation Using Curcumin as a Model Antioxidant for Oocyte Maturation

**DOI:** 10.3390/ani15223295

**Published:** 2025-11-14

**Authors:** Zhao Namula, Takeshige Otoi, Theerawat Tharasanit, Kaywalee Chatdarong, Megumi Nagahara, Oky Setyo Widodo, Aya Nakai, Suong Thi Nguyen, Yuichiro Nakayama, Maki Hirata, Fuminori Tanihara

**Affiliations:** 1 College of Coastal Agricultural Sciences, Guangdong Ocean University, Zhanjiang 524091, China; na_mu_la@126.com (Z.N.); nagahara@tokushima-u.ac.jp (M.N.); mhirata@tokushima-u.ac.jp (M.H.); tanihara@tokushima-u.ac.jp (F.T.); 2 Faculty of Veterinary Medicine, Universitas Airlangga, Surabaya 60115, Indonesia; oky.widodo@fkh.unair.ac.id; 3 Faculty of Veterinary Science, Chulalongkorn University, Bangkok 10330, Thailand; theerawat.t@chula.ac.th (T.T.); kaywalee.c@chula.ac.th (K.C.); 4 Bio-Innovation Research Center, Tokushima University, Tokushima 779-3233, Japan; nakai.aya.1@tokushima-u.ac.jp (A.N.); nguyensuongdadt@gmail.com (S.T.N.); yuichironakayama0@gmail.com (Y.N.)

**Keywords:** curcumin, meiotic competence, microdroplet, porcine oocyte, single culture system

## Abstract

Since individual embryos cannot be evaluated in group culture, establishing a single culture from in vitro maturation to in vitro culture may provide new insights into oocyte and embryo quality. This study aimed to develop a single culture system for individual oocytes, from in vitro maturation through fertilization to embryo development. The effects of curcumin supplementation during in vitro maturation on oocyte maturation, embryo development, and embryo quality were examined in single and group culture systems. Porcine oocytes were cultured individually in 20 µL microdroplets, with one oocyte per droplet, or in groups of 50 oocytes per 500 µL. The maturation medium contained curcumin at concentrations of 20 µM or less. Supplementation with 10 µM curcumin increased oocyte maturation in both systems compared to the controls. The fertilization rates and oocyte/embryo quality did not differ among the treatment groups. Oocytes matured with 10 µM curcumin in a single culture showed a higher blastocyst formation rate (7.0%) than the control (2.3%). In the group culture, 10 µM curcumin increased cleavage rates compared to the control (75.2% vs. 63.0%), but blastocyst formation rates did not differ. Blastocyst formation rates were similar between single and group cultures under control (2.3% and 4.3%, respectively) or 10 µM curcumin (7.0% and 11.4%, respectively) conditions. Therefore, porcine oocytes can develop to the blastocyst stage in a single culture system. Incorporating antioxidants during in vitro maturation may be an effective condition for in vitro embryo culture that can be implemented in a single oocyte.

## Simple Summary

This study developed a single culture system for individual oocytes from in vitro maturation through fertilization to embryo development, examining the effects of curcumin supplementation. When porcine oocytes were matured individually in microdroplets with 10 µM curcumin, supplementation increased maturation rates and improved blastocyst formation rates. Moreover, the maturation and blastocyst formation rates of oocytes matured individually in microdroplets were similar to those of oocytes matured in groups when oocytes were matured in the presence or absence of 10 µM curcumin. The single culture system enabled the development of porcine oocytes to the blastocyst stage, although at a limited rate. Furthermore, the incorporation of antioxidants, such as curcumin, during maturation appeared to enhance oocyte maturation and developmental potential under these conditions.

## 1. Introduction

In mammalian in vitro culture systems, oocyte and embryo development is influenced by various factors, including oxidative stress, medium composition, and cell–cell interactions. Individual culture systems often reduce embryo development because of decreased paracrine support compared to group culture conditions [1,2,3]. However, individual or single-oocyte culture systems have been developed to precisely evaluate oocyte quality and eliminate variability caused by intercellular influences [4,5]. The establishment of a culture system for individual oocytes or embryos can provide more accurate information on protein and DNA synthesis, oxygen consumption, and nutritional status. In pigs, especially, the establishment of the single culture system for the entire process, from initiating in vitro maturation (IVM) to in vitro culture (IVC), may provide new insight into intrinsic oocyte and embryo quality, potentially leading to the identification of new quality parameters. However, to our knowledge, no report has described a complete single-oocyte culture system covering the entire process, from initiating IVM through in vitro fertilization (IVF) to IVC in pigs.

Oxidative stress induces structural and functional damage, resulting in adverse effects on oocyte maturation and embryo development, such as apoptosis and DNA damage [6,7]. It has been reported that supplementation with antioxidants, such as ergothioneine [8], reduces reactive oxygen species (ROS)-induced oxidative stress and improves oocyte meiosis and embryonic development. Curcumin, a hydrophobic polyphenolic compound derived from the rhizome of *Curcuma longa*, acts as an antioxidant to reduce ROS levels, thereby decreasing lipid peroxide levels and mitigating H_2_O_2_-induced oxidative damage [9]. Our previous study using the group culture system demonstrated that curcumin protects oocytes by reducing DNA fragmentation and increasing oocyte maturation and embryonic development [10]. Other studies have also shown that curcumin protects oocytes from oxidative damage [11,12]. However, information on whether curcumin supplementation improves the rates of maturation and fertilization and the developmental competence of porcine oocytes, when cultured using a single culture system, is lacking.

This study aimed to establish a single culture system for individual oocytes from IVM through IVF to blastocyst formation. In addition, the effects of curcumin supplementation during IVM on oocyte maturation, embryo development, and oocyte and embryo quality were investigated in a single culture system. In this study, research-grade curcumin was used to investigate its effects to ensure reproducibility and avoid the variability often associated with food-grade turmeric extracts.

## 2. Materials and Methods

### 2.1. Ethical Approval

Porcine ovaries were obtained from a local slaughterhouse. No animals were sacrificed specifically for the purposes of this study.

### 2.2. In Vitro Maturation (IVM)

Oocyte collection and IVM were performed as previously described by Namula et al. [10] with minor modifications. Briefly, ovaries of prepubertal crossbred gilts (Landrace × Large White × Duroc) were collected from a local slaughterhouse. During this winter, when the outside temperature ranged from approximately 3 °C to 12 °C, the ovaries were transported to the laboratory in pre-warmed 0.9% saline (approximately 35 °C) within 2 h of slaughter. Cumulus-oocyte complexes (COCs) were collected from follicles (3–6 mm in diameter) using a surgical blade. Only COCs with uniform dark-pigmented ooplasm and intact cumulus cell mass were selected for this experiment. The COCs were placed in microdroplets of IVM medium (one oocyte per 20 µL medium) in 35 mm Petri dishes (Falcon 1008; Becton Dickinson, Lincoln Park, NJ, USA) containing 30–35 microdroplets under mineral oil (Sigma-Aldrich, St. Louis, MO, USA). The IVM medium consisted of TCM-199 with Earle’s salts (Thermo Fisher Scientific, Waltham, MA, USA), supplemented with 10% (*v*/*v*) porcine follicular fluid, 0.6 mM cysteine, 50 µM sodium pyruvate, 2 mg/mL d-sorbitol (Wako Pure Chemical Industries Ltd., Osaka, Japan), 10 IU/mL equine chorionic gonadotropin (eCG, Kyoritsu Seiyaku, Tokyo, Japan), 10 IU/mL human chorionic gonadotropin (hCG, Kyoritsu Seiyaku), and 50 µg/mL gentamicin. The COCs were individually cultured in IVM medium supplemented with hormones (eCG and hCG) for the first 22 h and subsequently cultured in IVM medium without hormones for an additional 22 h at 39 °C in a humidified incubator containing 5% CO_2_. To assess the effect of curcumin supplementation on the meiotic competence and DNA integrity of oocytes, as well as on fertilization and blastocyst formation, the COCs were individually cultured in IVM medium containing curcumin (≥94% HPLC grade, Sigma-Aldrich; Cat. No. C7727) dissolved in dimethyl sulphoxide and diluted to 0, 5, 10, and 20 µM before use in each experiment. As a control, COCs were cultured in a maturation medium without curcumin and a dilution solution (dimethyl sulphoxide). Moreover, to compare the single-culture microdroplet system with the standard group culture, approximately 50 COCs were cultured in 500 µL maturation medium containing either 0 or 10 µM curcumin, overlaid by mineral oil in a 4-well dish (Thermo Fisher Scientific) under the same conditions, a concentration previously reported to improve maturation and developmental competence of porcine oocytes in the group culture system [10].

### 2.3. Analysis of Oocyte Nuclear Maturation and DNA Fragmentation

To determine nuclear maturation and DNA fragmentation, the oocytes were fixed after IVM and analyzed using a combined technique of simultaneous nuclear staining and terminal deoxynucleotidyl transferase nick-end labeling (TUNEL), as previously described by Nagahara et al. [8]. Briefly, oocytes were denuded of cumulus cells using 150 IU of hyaluronidase and mechanical pipetting. Denuded oocytes were fixed in 4% paraformaldehyde at 4 °C overnight, permeabilized with 0.1% Triton-X100 for 40 min at room temperature, and then incubated in fluorescein-conjugated 2-deoxyuridine-5-triphosphate (TUNEL reagent; Roche Diagnostics Corp., Tokyo, Japan) for 1 h at 38.5 °C. After TUNEL staining, oocytes were counterstained with 1 µg/mL 4′,6-diamidino-2-phenylindole (DAPI; Thermo Fisher Scientific). The labeled oocytes were examined using an epifluorescence microscope fitted with epifluorescence illumination. The oocytes were determined to be either in the germinal vesicle (GV), condensed chromatin (CC), metaphase I (MI), anaphase I to telophase I (AT), or metaphase II (MII) stage according to the chromatin configuration based on DAPI staining. Unidentifiable chromatin configurations were classified as degenerated (DG). To assess DNA damage, all oocytes collected after IVM in each treatment group were examined, and nuclei labeled with TUNEL were counted.

### 2.4. In Vitro Fertilization, In Vitro Culture, and Assessment

In vitro fertilization (IVF) was performed as previously described by Namula et al. [10] with minor modifications. Semen was collected from three mature, fertile Large White boars using the gloved-hand method. After dilution with modified Modena extender, the semen was pooled and cryopreserved according to the protocol described by Namula et al. [13]. Briefly, semen was cooled to 5 °C and frozen in 0.25 mL straws using a two-step glycerol-based extender. On the day of IVF, the straws were thawed by immersion in a 38 °C water bath for 10 s. Thawed spermatozoa were transferred into 5 mL porcine fertilization medium (PFM; Research Institute for the Functional Peptides Co., Yamagata, Japan) and washed by centrifuging at 550× *g* for 5 min. Frozen-thawed spermatozoa were centrifuged and resuspended directly in fertilization medium. After centrifugation, the sperm pellet was resuspended in PFM to achieve a concentration of 3.0 × 10^6^ cells/mL. The spermatozoa (10 µL) were added to microdroplets of 10 µL PFM containing one oocyte under mineral oil in the Petri dishes for individual culture (one oocyte per 20 µL medium). The final sperm concentration was adjusted to 1.5 × 10^6^ cells/mL. The oocytes were co-incubated for 5 h at 39 °C in a humidified incubator containing 5% CO_2_, 5% O_2_, and 90% N_2_. After co-incubation, the presumptive zygotes were denuded of the cumulus cells and attached spermatozoa by mechanical pipetting. Dendued zygotes were subsequently cultured in microdroplets of 20 µL porcine zygote medium (PZM-5; Research Institute for the Functional Peptides Co.) overlaid by mineral oil in the Petri dishes (one zygote per 20 µL medium) at 39 °C in a humidified incubator with an atmosphere of 5% CO_2_, 5% O_2_, and 90% N_2_. To compare the single-culture microdroplet system with the standard group culture, approximately 50 oocytes matured in the presence or absence of 10 µM curcumin were fertilized in PZM (500 µL) overlaid by mineral oil in a 4-well dish under the same conditions.

To evaluate fertilization, some zygotes were fixed at 10 h after insemination with acetic acid: ethanol (1:3 *v*/*v*) for 48–72 h. The fixed zygotes were stained with acetic orcein (1% orcein in 45% acetic acid) and examined using phase-contrast microscopy. The zygotes containing female and male pronuclei were considered as fertilized and categorized as normal or polyspermic based on the number of swollen sperm heads and/or pronuclei in the cytoplasm [10].

Only cleaved embryos were selected and recorded 72 h after IVF, and transferred to microdroplets of 20 µL porcine blastocyst medium (PBM; Research Institute for the Functional Peptides Co.) overlaid by mineral oil in the Petri dishes (one embryo per 20 µL medium). The embryos were then cultured for an additional four days to assess their development into blastocysts. After blastocyst formation, DNA fragmentation was evaluated using the TUNEL assay, as previously described. DNA fragmentation indices were calculated by dividing the number of cells containing DNA-fragmented nuclei (labeled with TUNEL) by the total number of cells. To compare the single-culture microdroplet system with the standard group culture, the embryos were cultured in each culture medium (500 µL) overlaid by mineral oil in a 4-well dish under the same conditions.

### 2.5. Statistical Analyses

The experiments were repeated four times for oocytes that were matured with curcumin. Percentage data were subjected to arcsine transformation before statistical analysis. Statistical significance was evaluated by one-way analysis of variance (ANOVA), followed by the Games–Howell post hoc test using STATVIEW 4.0 (Abacus Concepts, Inc., Berkeley, CA, USA). Statistical significance was set at *p* < 0.05.

## 3. Results

As presented in Table 1, no differences were observed in the rates of oocytes remaining at the GV stage among the treatment groups in both single- and group-culture systems. In the single culture, the maturation rates of oocytes cultured with 5 and 10 µM curcumin in the microdroplets were significantly higher (*p* < 0.05) than those of control oocytes matured without curcumin and a dilution solution. Similarly, in the group culture, supplementation with 10 µM curcumin significantly increased the rate of MII-stage oocytes compared with the control without curcumin (*p* < 0.05). Maturation rates did not differ between the single and group culture systems for oocytes matured under the control conditions or with 10 µM curcumin. In both culture systems, no differences were observed in the proportions of oocytes with DNA-fragmented nuclei after maturation among the treatment groups.

As presented in Table 2, no significant differences were observed in the total and monospermic fertilization rates among the treatment groups within each culture system. In the single culture, the blastocyst formation rate of oocytes matured with 10 µM curcumin was significantly higher (*p* < 0.05) than that of oocytes matured without curcumin, with a dilution solution, or with 20 µM curcumin. In the group culture, although the cleavage rate of oocytes matured with 10 µM curcumin was significantly higher (*p* < 0.05) than that of control oocytes, there was no difference in the blastocyst formation rate between the two treatment groups. Blastocyst formation rates did not differ between the single and group culture systems for oocytes matured under control conditions or with 10 µM curcumin. In both culture systems, no differences were observed in the total cell number and DNA fragmentation index of blastocysts among the treatment groups.

## 4. Discussion

This study aimed to establish a single culture system for individual oocytes, particularly during whole in vitro culture from the start of IVM using an antioxidant (curcumin). Our results demonstrated that oocytes cultured individually during IVM, IVF, and IVC can effectively develop to the blastocyst stage. Moreover, the curcumin supplementation during IVM increased the meiotic competence and improved the blastocyst formation rate. This approach may be particularly beneficial for reproductive applications involving limited oocyte availability, such as in genetically modified pigs or rare-breed animals, beyond evaluating the individual metabolic capacity of oocytes. Culturing embryos from only a few oocytes could support genetic management and future breeding strategies. Therefore, our results may offer insights into embryo production systems where only a limited number of oocytes are available.

While the blastocyst rates observed in this study were relatively low (maximum 7.0% with 10 µM curcumin), this limitation may not be uncommon in single-embryo cultures, where the lack of group effects reduces developmental competence [3,14]. In vitro culture of oocytes and embryos individually typically results in low blastocyst rates and reduced embryo quality [3,14,15]. In this study, however, the blastocyst formation rate of oocytes cultured using a single culture system without curcumin supplementation was 2.3%, which was similar to that of oocytes matured by the group culture system (4.3%). In standard practice, IVM and embryo culture are usually performed in group systems, which are suggested to enhance developmental outcomes due to paracrine interactions [3,14]. Our previous study also demonstrated a reduced developmental competence of bovine embryos in single culture compared to group culture [1]. These observations are consistent with several reports in some species, including humans and bovines. Group culture of human embryos significantly improved blastocyst formation and clinical outcomes compared to individual culture, due to the beneficial effects of accumulated autocrine and paracrine factors [2]. Similarly, bovine embryos cultured in groups had a higher blastocyst formation rate than those cultured individually, indicating the importance of embryo–embryo communication [3]. In this study, however, no significant differences were observed in the maturation and blastocyst formation rates between the single and group culture systems when oocytes were matured in the presence or absence of 10 µM curcumin. One possible explanation is the inherently low developmental competence of the oocytes used in this study, as reflected by the overall low blastocyst formation rates in both conditions (2.3% and 4.3%). Under such suboptimal conditions, the paracrine/autocrine benefits of group culture may not be sufficient to improve outcomes. Conversely, Travaglione et al. [16] demonstrated that bovine zygotes can develop normally to the blastocyst stage even in a minimal culture environment (approximately 70 nL microwells). They suggested that the construction of a closed microenvironment mitigates the adverse effects associated with individual cultures and maintains developmental potential. Another possibility is that the confined microdroplet system used in the present study may have partially reproduced this favorable condition by limiting the diffusion of autocrine factors, thereby maintaining oocyte maturation and subsequent embryo development.

Yuan et al. [17] demonstrated improved developmental rates using three cytokines (FGF2, LIF, and IGF1)-supplemented maturation medium (FLI medium) in the group culture system. Although this study does not directly compare with FLI medium or batch cultures, it explores the potential of curcumin as an antioxidant to support oocyte development under single-culture conditions. In this study, supplementation of 10 µM curcumin during IVM significantly positively affected the development rates of porcine oocytes in the single culture system. While the differences in the development rates of oocytes after IVF between the groups were statistically significant, the absolute numbers remained modest. These outcomes might be influenced by variability (e.g., batch of the slaughterhouse ovaries, age and diet of the slaughtered animals, and others) in the oocyte source. However, this result is consistent with the finding of Namula et al. [10], who demonstrated that the addition of 10 µM curcumin during IVM in the group culture improved oocyte development to the blastocyst stage. Moreover, we found that there was no significant difference in the blastocyst formation rates of oocytes cultured in the group culture, but the value of oocytes matured with 10 µM curcumin was more than twice that of control oocytes. Standard in vitro conditions fundamentally contain oxygen levels approximately three times higher than those within the lumen of the female reproductive tract, resulting in increased free radical production [18,19]. In group culture systems, oocytes and cumulus cells consume oxygen and secrete paracrine factors, reducing oxidative stress [6,20]. This protective effect is absent in single oocyte cultures due to the lack of neighboring cells, making oocytes more susceptible to reactive oxygen species. Therefore, antioxidant supplementation in single-oocyte cultures, such as with curcumin, may compensate for the lack of intercellular interactions and further clarify its effects compared with the group culture. An imbalance between ROS and its defense mechanism affects the in vitro maturation and fertilization ability of oocytes [21]. Curcumin and its derivatives exhibit antioxidant properties to scavenge free radicals [22]. Moreover, the antioxidant action of curcumin prevents oxidative stress and apoptosis by inhibiting methylglyoxal in embryonic stem cells and blastocysts in mice [23]. In this study, curcumin supplementation (10 μM) during IVM did not affect fertilization rates but improved oocyte maturation as well as embryo development. Although we could not directly measure intracellular ROS levels in this study, the observed improvements in blastocyst formation rates were consistent with previous reports on the effects of other antioxidants in porcine oocytes [11,24]. As the in vitro oocyte culture condition lacks external non-enzymatic antioxidant protection, curcumin supplementation as external protection in the single culture system may be an effective strategy in vitro. In this study, we observed that curcumin supplementation did not increase the total blastocyst cell number or reduce DNA fragmentation in blastocysts in either single or group culture systems. Many studies have examined TUNEL positivity rates and total cell numbers to evaluate the quality of in vitro-fertilized porcine embryos. Although TUNEL-positive nuclei have been observed in approximately 10% of blastocysts in the group culture system [10,25], no cut-off value for high fragmentation has been defined. Thus, the observed improvement may primarily reflect an increased proportion of embryos reaching the blastocyst stage rather than a genuine enhancement of embryo quality.

In conclusion, this study demonstrates that porcine oocytes can be cultured individually from IVM to the blastocyst stage. Supplementation with 10 µM curcumin improved oocyte maturation and blastocyst formation in this setting. While the developmental rates were limited, the findings suggest that antioxidant supplementation may be beneficial in single-culture systems, particularly under conditions where only a small number of oocytes are available. To our knowledge, this is the first study to report the complete in vitro development of porcine oocytes cultured individually from IVM to the blastocyst stage. Previous studies have focused on group cultures or have not tracked development from IVM to the blastocyst stage in a single culture [10,11,12].

Several limitations and future research directions should be considered to improve the reproducibility and applicability of this system. These include the following points: In this study, since each oocyte was cultured in individual 20 µL microdroplets in the same dish, the possibility of metabolite diffusion through the shared oil layer covering the droplets cannot be ruled out. Therefore, the current system may not completely reproduce a “single culture” environment in the strict sense. Furthermore, since the effect of drop size on development has been reported in single-culture systems [26,27], it may be necessary to optimize drop size to maximize developmental potential in future applications. This study evaluated the effects of antioxidant supplementation during IVM using a single-oocyte culture system. While only curcumin was tested, future research should examine other antioxidants and explore the applications of a single-oocyte system. Moreover, porcine follicular fluid (pFF) was used as a supplement in the IVM medium, as it is commonly used in porcine embryo culture systems and supports oocyte competence. However, pFF introduces undefined biological variables. Future studies should consider using chemically defined supplements, such as polyvinyl alcohol (PVA), to reduce variability when evaluating antioxidant mechanisms.

## 5. Conclussions

We established a single-oocyte culture system that enabled porcine oocytes to develop from the IVM to the blastocyst stage. Curcumin (10 µM) enhanced oocyte maturation and embryonic development. Despite modest developmental outcomes, our findings indicate that antioxidants can support the culture of individual oocytes, providing a platform for studies when a limited number of oocytes are available.

## Figures and Tables

**Table 1:** Effects of curcumin supplementation in the maturation medium on the meiotic competence of porcine oocytes cultured by the single- and group-culture systems *.

Curcumin (µM)		No. of Examined Oocytes	No. (%) of Oocytes with **						
			GV	CC	M I	AT	M II	DG	DNA-Fragmented Nuclei ***
Single culture	Control	105	2 (1.6 ± 0.9)	9 (8.4 ± 2.1)	21 (22.1 ± 4.6) ^a,b^	6 (5.0 ± 1.7)	65 (61.2 ± 1.7) ^c,b^	2 (1.6 ± 0.9)	11 (11.9 ± 3.5)
	0	109	4 (4.1 ± 1.6)	14 (12.0 ± 1.8)	20 (20.8 ± 5.0) ^a,b^	1 (0.8 ± 0.8)	67 (60.0 ± 4.5) ^a,b,c^	3 (2.4 ± 1.5)	8 (9.3 ± 4.1)
	5	103	2 (1.7 ± 1.0)	12 (11.1 ± 4.3)	12 (13.3 ± 4.9) ^a,b^	0 (0)	77 (74.0 ± 1.8) ^a^	0 (0)	4 (5.5 ± 3.4)
	10	104	2 (1.6 ± 0.9)	5 (5.2 ± 2.2)	9 (9.6 ± 2.8) ^b^	3 (2.5 ± 0.8)	83 (79.4 ± 1.7) ^a^	2 (1.7 ± 1.7)	1 (0.8 ± 0.8)
	20	105	2 (1.7 ± 1.7)	10 (9.2 ± 1.6)	15 (16.3 ± 4.4) ^a,b^	1 (1.8 ± 1.8)	74 (68.6 ± 4.4) ^a,b,c^	3 (2.5 ± 1.6)	2 (1.7 ± 1.7)
Group culture	Control	97	4 (3.9 ± 1.6)	6 (7.1 ± 2.7)	28 (28.9 ± 1.2) ^a^	2 (2.0 ± 2.0)	53 (54.3 ± 2.0) ^c^	4 (3.8 ± 2.3)	12 (12.4 ± 2.4)
	10	115	3 (2.5 ± 1.5)	8 (7.0 ± 1.5)	14 (10.6 ± 3.2) ^b^	0 (0)	85 (75.5 ± 3.5) ^a,b^	5 (4.4 ± 3.0)	5 (3.9 ± 1.6)

* Four replicate trials were conducted. Percentages are expressed as mean ± SEM. In a single culture, the oocyte was cultured in multiple microdroplets covered by mineral oil in the same dish (one oocyte per 20 µL). In the group culture, the oocytes were cultured in a 4-well dish (50 oocytes per 500 µL). ** They were classified as “germinal vesicle (GV),” “condensed chromatin (CC),” “metaphase I (MI),” “anaphase I/telophase I (AT)” or “metaphase II (MII)”. Unidentifiable chromatin configurations were classified as “degenerated (DG)”. *** The DNA-fragmented nuclei were defined as the ratio of the number of oocytes containing an apoptotic nucleus to the total number of examined oocytes. DNA fragmentation in nuclei was observed only in the classified oocytes with the chromatin configuration. ^a–c^ Values with different superscript letters in the same column are significantly different (*p*  <  0.05).

**Table 2:** Effects of curcumin supplementation in the maturation medium on the fertilization and development of porcine oocytes by the single- and group-culture systems *.

Curcumin (µM)		No. of Examined Oocytes	No. (%) of Oocytes **		No. of Examined Oocytes	No. (%) of Embryos		Total Cell Number of the Blastocyst	DNA-Fragmentation Index ***
			Fertilized	Monospermy		Cleaved	Developed to Blastocysts		
Single culture	Control	106	33 (34.4 ± 6.5)	28 (83.7 ± 9.3)	264	147 (55.8 ± 2.1) ^b^	6 (2.3 ± 0.3) ^c^	40.9 ± 1.0	18.5 ± 4.5
	0	104	25 (30.5 ± 12.1)	23 (93.8 ± 6.3)	257	129 (50.5 ± 2.3) ^b^	4 (1.4 ± 0.6) ^c^	37.7 ± 2.2	18.4 ± 2.1
	5	102	39 (43.7 ± 9.7)	33 (83.4 ± 7.0)	256	174 (67.0 ± 7.3) ^a,b^	10 (3.8 ± 0.8) ^b,c^	40.3 ± 3.2	17.9 ± 5.0
	10	102	42 (46.2 ± 9.0)	34 (80.4 ± 2.0)	256	193 (74.5 ± 7.0) ^a,b^	18 (7.0 ± 0.6) ^a,b^	41.3 ± 1.9	12.5 ± 3.8
	20	103	32 (34.3 ± 6.8)	26 (81.2 ± 9.0)	251	155 (62.4 ± 6.5) ^a,b^	6 (2.4 ± 0.4) ^c^	41.0 ± 2.4	11.1 ± 1.4
Group culture	Control	93	41 (43.9 ± 2.9)	29 (70.3 ± 2.6)	232	146 (63.0 ± 1.5) ^b^	10 (4.3 ± 0.6) ^a,c^	37.7 ± 1.3	14.8 ± 0.8
	10	95	52 (56.2 ± 3.4)	37 (71.2 ± 3.9)	226	170 (75.2 ± 2.4) ^a^	26 (11.4 ± 1.6) ^a^	37.8 ± 2.3	10.4± 0.7

* Four replicate trials were conducted. Percentages are expressed as mean ± SEM. In a single culture, the oocyte was cultured in multiple microdroplets covered by mineral oil in the same dish (one oocyte per 20 µL). In the group culture, the embryos were cultured in a 4-well dish (<50 embryos per 500 µL). ** The proportions of monospermic fertilization were calculated by dividing the number of monospermic fertilized oocytes by the total number of fertilized oocytes. *** The DNA fragmentation indices were defined as the ratio of the number of cells containing a DNA-fragmented nucleus and the total number of cells in a blastocyst. ^a–c^ Values with different superscript letters in the same column are significantly different (*p*  <  0.05).

## Data Availability

The data used to support the findings of this study have been included in this article.

## Abbreviations

The following abbreviations are used in this manuscript:

CC	Condensed chromatin
COC	Cumulus-oocyte complexes
DAPI	4′,6-diamidino-2-phenylindole
GV	Germinal vesicle
IVC	In vitro culture
IVF	In vitro fertilization
IVM	In vitro maturation
PBM	Porcine blastocyst medium
PFM	Porcine fertilization medium
PLSD	Protected least significant difference
PZM	Porcine zygote medium
ROS	Reactive oxygen species
